# Single-sample gene set enrichment analysis reveals the clinical implications of immune-related genes in ovarian cancer

**DOI:** 10.3389/fmolb.2024.1426274

**Published:** 2024-08-05

**Authors:** Weiwei Gong, Mingqin Kuang, Hongxi Chen, Yiheng Luo, Keli You, Bin Zhang, Yueyang Liu

**Affiliations:** ^1^ Department of Hematology and Oncology, Guangzhou Women and Children’s Medical Center, Guangzhou Medical University, Guangzhou, China; ^2^ Gynecology and Oncology Department of Ganzhou Cancer Hospital, Ganzhou, Jiangxi, China; ^3^ Department of Gynecology, Guangdong Provincial People’s Hospital (Guangdong Academy of Medical Sciences), Southern Medical University, Guangzhou, Guangdong, China; ^4^ The First Clinical Medical College, Southern Medical University, Guangzhou, China

**Keywords:** ovarian cancer, tumor immune microenvironment, immunotherapy, prognosis, immune checkpoint

## Abstract

**Purpose:**

Ovarian cancer (OC) is a common gynecological malignancy with poor prognosis and substantial tumor heterogeneity. Due to the complex tumor immune microenvironment (TIME) among ovarian cancer, only a few patients have an immune response to immunotherapy. To investigate the differences in immune function and identify potential biomarkers in OC, we established a prognostic risk scoring model (PRSM) with differential expression of immune-related genes (IRGs) to identify critical prognostic IRG signatures.

**Methods:**

Single-sample gene set enrichment analysis (ssGSEA) was used to investigate the infiltration of various immune cells in 372 OC patients. Then, COX regression analysis and Lasso regression analysis were used to screen IRGs and construct PRSM. Next, the immunotherapy sensitivity of different risk groups regarding the immune checkpoint expression and tumor mutation burden was evaluated. Finally, a nomogram was created to guide the clinical evaluation of the patient prognosis.

**Results:**

In this study, 320 immune-related genes (IRGs) were identified, 13 of which were selectively incorporated into a Prognostic Risk Scoring Model (PRSM). This model revealed that the patients in the high-risk group were characterized as having poorer prognosis, lower expression of immune checkpoints, and decreased tumor mutation load levels compared with those in the low-risk group. The nomogram based on the risk score can distinguish the risk subtypes and individual prognosis of patients with OC. Additionally, M1 macrophages may be the critical target for immunotherapy in OC patients.

**Conclusion:**

With the in-depth analysis of the immune microenvironment of OC, the PRSM was constructed to predict the OC patient prognosis and identify the subgroup of the patients benefiting from immunotherapy.

## Introduction

Ovarian cancer (OC) is one of the malignant tumors of female reproductive system, with enormous tumor heterogeneity (Vázquez-García et al., 2022; Olbromski et al., 2023). According to statistics from the American Cancer Society, there will be 19,710 new cases of OC among women and an estimated death of 13,270 in the United States in 2023, ranking fifth after lung cancer, breast cancer, colon cancer, and pancreatic cancer, seriously affecting women’s health (Siegel et al., 2023). OC has the most histological types of solid tumors, which can be divided into four subtypes, namely epithelial tumors, germ cell tumors, sex cord-stromal tumors, and metastatic tumors. The above-mentioned subtypes have different risk factors, cellular origin, molecular composition, clinical characteristics, and treatment (Matulonis et al., 2016). At present, the most commonly used treatment for OC is cytoreductive surgery combined with platinum-based standard chemotherapy. Nevertheless, a significant proportion of OC patients relapse shortly after the standard treatment regimen, and additionally, some even relapse during the ongoing course of chemotherapy. As recurrence is the leading cause of death in OC patients, the effectiveness of the current immunotherapy is not satisfactory (Armstrong et al., 2022). The clinical trials for immunotherapy in ovarian cancer have been marred by several predominant factors, including the inherent diversity of the immune microenvironment (Luo et al., 2024), the suppressive influence of regulatory T cells which can stifle therapeutic responses, the emergence of drug resistance that hampers long-term efficacy, the complexity introduced by tumor heterogeneity affecting patient responses variably (An and Yang, 2021), and the yet-to-be-fully-realized potential of combination therapies that aim to synergize for improved patient outcomes (Hoogstad-van Evert et al., 2020). For example, in the JAVELIN Ovarian 200 Phase III clinical trial (Zamarin et al., 2020), researchers enrolled a total of 566 patients with recurrent platinum-resistant or platinum-refractory ovarian, fallopian tube, or peritoneal cancer. These patients were randomly assigned to receive treatment with Avelumab and/or liposomal doxorubicin. The study results showed that the combination of Avelumab and liposomal doxorubicin did not extend the median progression-free survival (PFS), with median PFS being 3.5 months and 3.7 months, respectively. Additionally, this treatment approach did not improve the median overall survival (OS), with median OS being 13.1 months and 15.7 months, and the hazard ratio (HR) was 0.89 with a 95% confidence interval of 0.74–1.24. These data suggest that for patients with recurrent platinum-resistant or platinum-refractory ovarian cancer, the combination of chemotherapy with the anti-programmed death-ligand 1 (PD-L1) antibody (Avelumab) does not yield better clinical benefits. Therefore, an in-depth exploration of OC’s genetic differences and molecular functions may lead to new diagnostic, prognostic, and therapeutic biomarkers.

Tumor immune microenvironment (TIME) is a complex ecosystem composed of tumor cells, immune cells, cytokines, and other components. These elements have both tumor-promoting and anti-tumor effects, and their interactions determine the ultimate direction of tumor immune-related functions (Li et al., 2021; Harbin et al., 2023). The unrestricted proliferation of tumor cells will change TIME and evade immune surveillance by destroying the antigen presentation mechanism, strengthening the negative immune regulation pathway, recruiting tumor-promoting immune cells, and so on (Xu et al., 2022). Immunotherapy is mainly used to reshape TIME and restore the tumor-killing ability of anti-tumor immune cells. Given the exciting clinical benefits of immunotherapy in malignant tumors such as melanoma, clinical trials of immunotherapy in OC have emerged in the past few years. In a phase II randomized clinical trial called NRG, 100 patients with ovarian cancer were randomly divided into the Nivolumab group (49 cases, including 31 cases of platinum resistance), the Nivolumab and Ipimab group (51 cases, including 31 cases of platinum resistance). Within 6 months, the response rate of the combination group of Nivolumab and Epizumab was significantly higher (31.4% vs. 12.2%), and the median PFS was significantly longer (3.9 months vs. 2.0 months) than that of the single Navuliumab group (Zamarin et al., 2020). However, most of the immunotherapy clinical trials in OC had not achieved satisfactory results (Binnewies et al., 2018; Zhang et al., 2023). Moreover, OC has always been regarded as an immune-excluded tumor, which is characterized by insufficient T cell infiltration, low tumor mutation burden (TMB), poor antigen expression, and insensitive to the inherent killing of T cells (Hornburg et al., 2021). Hence, exploring the changes in TIME of OC and searching for immune biomarkers has been an issue that OC researchers have been trying to solve.

In this study, we explore potential immune biomarkers from the TIME perspective. Firstly, the single-sample gene set enrichment analysis (ssGSEA) was used to assess every OC sample from The Cancer Genome Atlas (TCGA). This method can effectively avoid the tumor heterogeneity of OC. Secondly, we aim to identify relevant gene enrichment pathways and obtain immune-related genes (IRGs) by comparing the gene expression differences between high- and low-immune groups. Then, the least absolute shrinkage and selection operator (Lasso) is used to select the most representative signatures from IRGs to build a prognostic risk scoring model (PRSM). And conduct external verification using a dataset in Gene Expression Omnibus data base (GEO). Finally, we try to find the reasons for poor prognosis in high-risk OC patients based on TMB levels, immune checkpoint expression levels, and immune cell correlation. In summary, 13 IRG signatures were generated to predict the risk subtypes, survival time, and immunotherapy response in OC patients. These findings offer valuable insights into the identification of immune biomarkers.

## Materials and methods

### Gene expression data source

First, The public transcriptome data and clinical data of OC were downloaded from TCGA database (https://portal.gdc.cancer.gov/). Here, 372 OC samples were embodied in total. Second, the GEO database (https://www.ncbi.nlm.nih.gov/geo/) provided GSE26712 dataset (n = 185 samples). Finally, TCGA data was utilized to filter IRGs and construct PRSM, while GEO data was employed as external data to verify the predictive accuracy of PRSM.

### ssGSEA

ssGSEA is a common method for immune cell infiltration analysis. ssGSEA estimates the relative abundance of different immune cell types in each sample by comparing the gene expression data of each sample with immune cell markers. Subsequently, according to the ssGSEA score, 372 OC patients were divided into a high immune group (n = 329) and a low immune group (n = 43).

### Difference of TME between high- and low-immunity groups

Tumor microenvironment (TME) refers to the Internal environment where tumor cells are generated and live, which is heterogeneous and consists of multiple cell types. According to the method published by Yoshihara et al. (2013) the ESTIMATE algorithm is used to estimate tumor purity. Generally speaking, the results of the ESTIMATE algorithm are divided into stromal score and immune score, representing the presence of stromal cells and immune cells, respectively. Adding two fractions together yields the estimated score, which represents the purity of the tumor.

### Difference of TIME between high- and low-immunity groups

CIBERSORT is a widely used computational method in tumor immunology research. With the help of R-packets, it can quantitatively analyze the proportion of different immune cell subsets in ovarian cancer tissue using gene expression data.

### Screening for IRGs

First of all, differential gene expression analysis was performed on high- and low-immune groups using the “limma” package in the R language to obtain differentially expressed genes (DEGs), with| FDR |> 1, *P* < 0.05 as the screening criteria. Next, immunity genes (IGs) were downloaded from the immport database (https://www.immport.org/shared/home). Finally, the intersection of DEGs and IGs is taken to obtain the IRGs.

### Gene enrichment analysis

With the help of R-package, kyoto encyclopedia of genes and genomes (KEGG) and gene set enrichment analysis (GSEA) were used to analyze the related signaling pathways of DEGs in high- and low-immune groups.

### Constructing protein-protein interaction networks

To start with, export IRGs separately as a dataset, and then import this dataset into the STRING database (https://cn.string-db.org/) for visual analysis to obtain protein-protein interaction networks (PPI networks). In PPI networks, different nodes or colour represent different protein interactions.

### Established a prognostic risk-scoring model

Firstly, Lasso regression analysis was used to identify key IRGs. Secondly, the risk score for each OC patient was calculated based on the correlation coefficients of key IRGs and key IRGs expression levels using the risk score calculation formula. Then, based on the median risk score as the optimal cutoff value, 372 OC samples were divided into high-risk and low-risk groups. Next, the Kaplan-Meier survival curve was used to compare the overall survival (OS) time differences between high- and low-risk groups. Finally, Receiver operating characteristic and calibration (ROC) curve and calibration curve were used to test the accuracy of PRSM. And the risk-scoring formula is as follows:

$$\text{Risk Score}=\sum_{i}^{n}\text{ coef }\left(i\right)\times \mathrm{Exp}\;\left(i\right)$$

In the formula of PRSM, coef represents the correlation coefficient of IRGs, Exp represents the expression of key IRGs, i represents the IRGs, and n represents the number of key IRGs.

### Verification of prognostic risk scoring model

Due to the high accuracy of PRSM in predicting OS in OC patients, we used the GSE26712 dataset from the GEO database as external data to verify the accuracy of PRSM’s OS prediction and construct a Kaplan Meier survival curve.

### Analysis of differences in immune checkpoints and tumor mutation burden

With the help of R package, we analyzed the expression differences of immune detection points such as PD1, PDL1, CTLA4 between high- and low-risk groups. Next, we analyzed the differences in TMB levels between high and low risk groups, and divided 372 OC patients in the TCGA database into high TMB and low TMB groups using the median TMB as the cutoff value. Then, survival curve analysis was performed on OC patients in the high and low TMB groups. Finally, prognostic analysis was conducted on 372 OC patients in the TCGA database by incorporating both risk scores and TMB levels.

### Establishment and evaluation of a nomogram

Firstly, univariate COX regression analysis was used to test whether the risk score is a risk factor for the prognosis of OC patients. Next, multivariate COX regression analysis was employed to test whether the risk score can serve as an independent prognostic risk factor for OC patients. Then, a nomogram was constructed based on patient’s age, tumor FIGO stage, histological grade, and PRSM risk score. And the nomogram was conducted to evaluate the total score, which predicting 1-year, 3-year, and 5-year survival rates. Finally, the calibration curve was utilized to verify the consistency of the nomogram outcome with real-world events.

### Statistical analysis

The main software and versions required for data analysis in this study are as follows: R language (4.1.3), Rstudio (2021.09.1 + 372), Strawberry perl (5.30.1), limma package (3.50.3), ggplot2 package (3.4.2), survival package (3.2.13), dplyr package (1.1.2), ggallivian package (0.12.5), caret package (6.0.94). Unless otherwise specified in the text, *P* < 0.05 is considered to have a statistical difference.

## Results

### Identification of two subtypes of immune infiltration in OC patients

**FIGURE 1:**
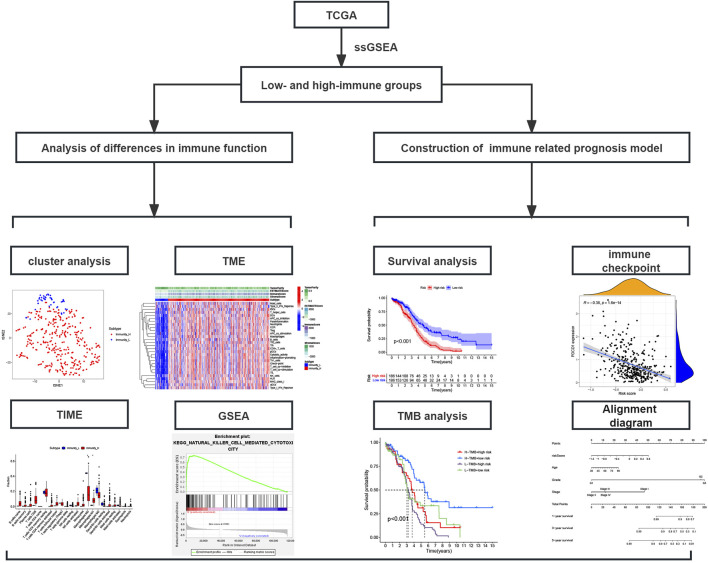
Study flowchart; TCGA, The Cancer Genome Atlas database; ssGSEA, Single-sample gene set enrichment analysis; TME, Tumor microenvironment; TIME, Tumor immune microenvironment; GSEA, Gene set enrichment analysis.

**FIGURE 2:**
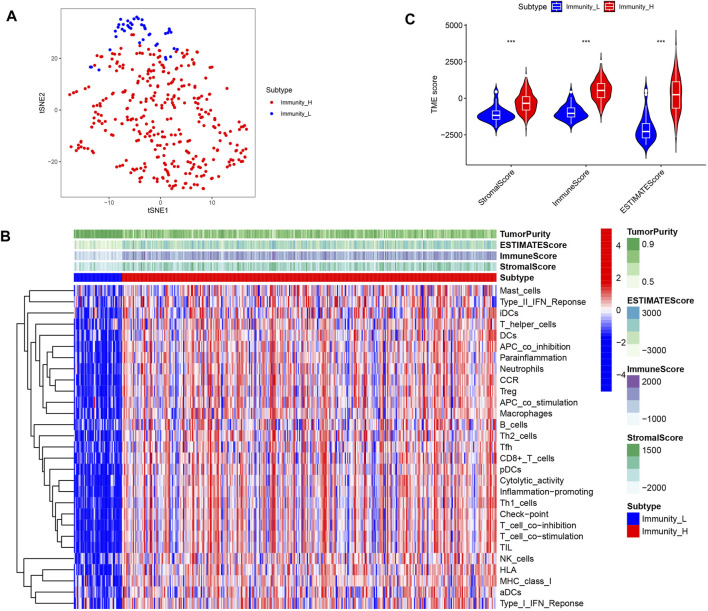
Identification of two subtypes of immune infiltration in OC patients. **(A)** tSNE describes the grouping between different immune subgroups. **(B)** Heat map of immune cell infiltration analysis between different immune subgroups. **(C)** Differences in the content of stromal cells and immune cells between immune subgroups.

In this study, in order to effectively avoid OC tumor heterogeneity, we chose the ssGSEA method to conduct bioinformatics analysis on 372 OC transcriptome data in the TCGA database. In this way, we explored the differences in immune-related functions among different OC patients and constructed a prognosis model based on IRG signatures (Figure 1). Based on the representative makers of 29 types of immune cells, we used the ssGSEA tool to perform immune infiltration analysis. Then, we successfully divided 372 OC patients into two subtypes, namely, the high-immune group (n = 329) and the low-immune group (n = 43). For such a large number of OC patients being assigned to the high-immune group, we believed there are certain doubts about the limited effectiveness of immunotherapy in clinical practice. At the same time, this result also aroused our extreme enthusiasm for further research. Consequently, we reduced the dimension of the distribution of the two groups of OC patients with the help of tSNE. And the results of tSNE showed that the high- and the low-immune groups of OC patients were really different in terms of immune cell infiltration (Figure 2A).

### Differences in TME between high- and low- immune groups

TME is mainly composed of stromal cells and immune cells. Firstly, we visualized the infiltration of 29 types of immune cells in two subgroups. We found that immune cells with anti-tumor effects, such as CD8^+^ T cells, Th1 cells and NK cells, showed significant infiltration in the high- immune group. However, cells such as Th2 cells and Treg cells that have tumor promoting and immunosuppressive effects were also highly invasive (Figure 2B). This reveals the complexity of TIME. Next, we used the ESTIMATE algorithm to score the high- and low- immune groups to evaluate the content of stromal cells and immune cells in OC. We found that the high- immune group had higher stromal cell scores and immune cell scores (Figure 2C). This meant that OC in the high-immune group have more stromal cells and immune cells.

### Differences in TIME and related signaling pathways between high- and low-immune groups

**FIGURE 3:**
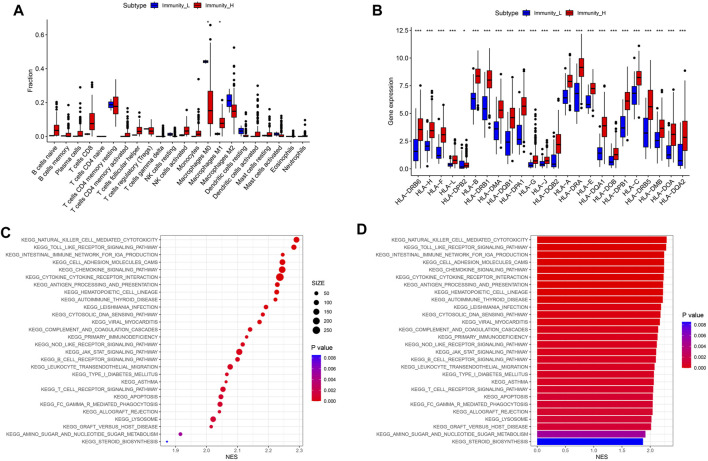
Differences in immune cell content and related signaling pathways between immune subgroups. **(A)** Boxplot of immune cell content in high and low risk groups. **(B)** Boxplot of HLA related gene expression. **(C)** KEGG analysis signal bubble chart. **(D)** KEGG analysis signal bar chart.

In order to further determine which type of cells are dominant in the high- and low-immune groups, we conducted an analysis of their immune cell content. The results showed that compared to the low- immune group, the high- immune group had a lower content of M0 macrophages in TIME, while the content of M1 macrophages with anti-tumor and immunomodulatory effects was significantly higher (Figure 3A, *P* < 0.05). In addition, human leukocyte antigens (HLAs) gene expression analysis indicated that the high-immune group has significant HLAs expression level (Figure 3B, *P* < 0.001). Next, we compared the differences in signaling pathways between high- and low- immune groups. We found significant enrichment in signaling pathways such as JAK/STAT signaling pathway, natural killer cell mediated cytotoxicity signaling pathway, and chemokine signaling pathway (Figures 3C, D). Among them, JAK/STAT signaling pathway was involved in regulating the polarization process of M1 macrophages.

### Active immune related signaling pathways in the high-immune group

**FIGURE 4:**
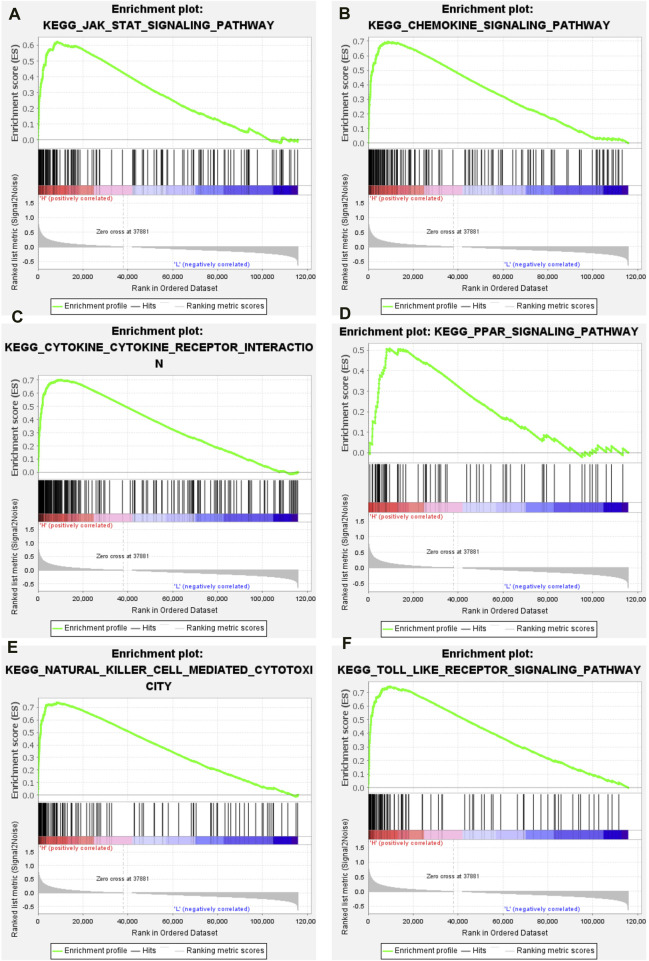
GSEA results of high immune group. **(A)** JAK/STAT signaling pathway. **(B)** Chemokine signaling pathway. **(C)** Cytokine-cytokine receptor interaction signaling pathway. **(D)** PPAR signaling pathway. **(E)** Natural killer cell mediated cytotoxicity signaling pathway. **(F)** Toll like receptor signaling pathway.

The GSEA package in the R language was used to explore the enriched signaling pathways in high-immune groups. The results showed that many immune related signaling pathways were significantly enriched in the high- immune group. These signal pathways include JAK/STAT signaling pathway (Figure 4A), chemokine signaling pathway (Figure 4B), cytokine-cytokine receptor interaction signaling pathway (Figure 4C), PPAR signaling pathway (Figure 4D), natural killer cell mediated cytotoxicity signaling pathway (Figure 4E), toll like receptor signaling pathway (Figure 4F).

### Construction of IRG signatures

**FIGURE 5:**
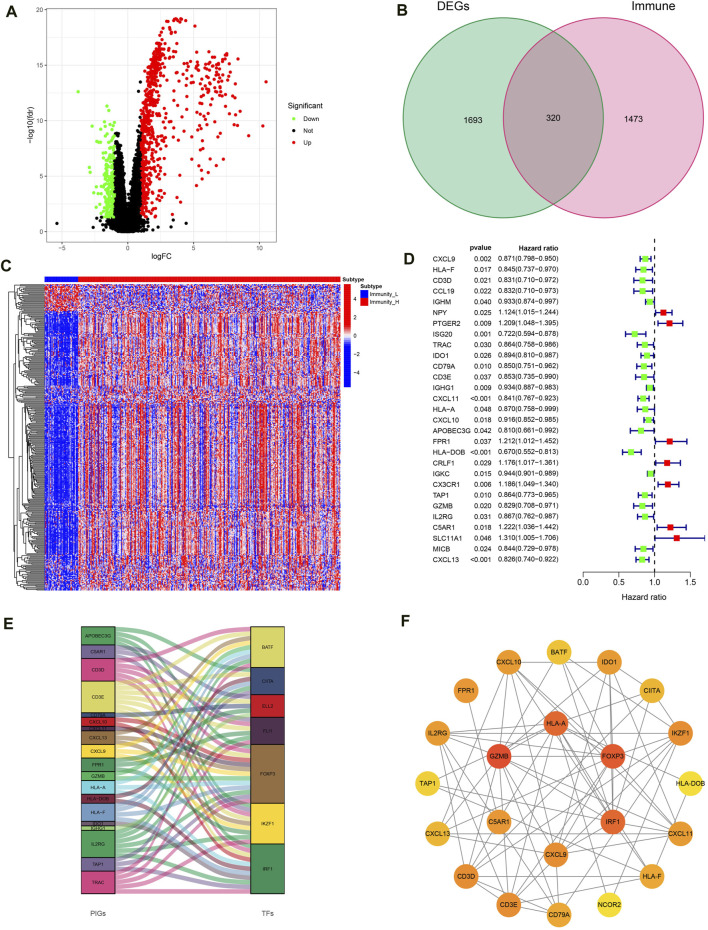
Construction of IRG signatures. **(A)** Volcano plot of DEGs. **(B)** Venn diagram of DEGs and IGs. **(C)** Differential expression heatmap of IRGs in high and low immune groups. **(D)** Forest map of prognosis related IRGs. **(E)** Sankey diagram of prognosis related IRGs and TF. **(F)** PPI network diagram of prognosis related IRGs.

With | FDR |> 1, *P* < 0.05 as the screening criteria, 2013 DEGs were identified by using the limma package in the R language to handle data of high- and low-immune groups (Figure 5A). Then, the DEGs were intersected with IGs obtained from the immport database, resulting in the identification of 320 IRGs and draw gene expression heatmaps (Figures 5B, C). Next, COX regression analysis was conducted to compare 320 IRGs with the total survival time and survival outcome of OC patients, and 29 IRGs related to OC prognosis were selected (Figure 5D). Finally, we conducted co-expression analysis of these prognosis related IRGs and transcription factors in an attempt to identify potential downstream key genes and construct a Sankey diagram (Figure 5E). Additionally, we conducted protein interaction analysis on prognosis related IRGs and obtained a PPI network diagram (Figure 5F).

### The evaluation and validation of PRSM

**FIGURE 6:**
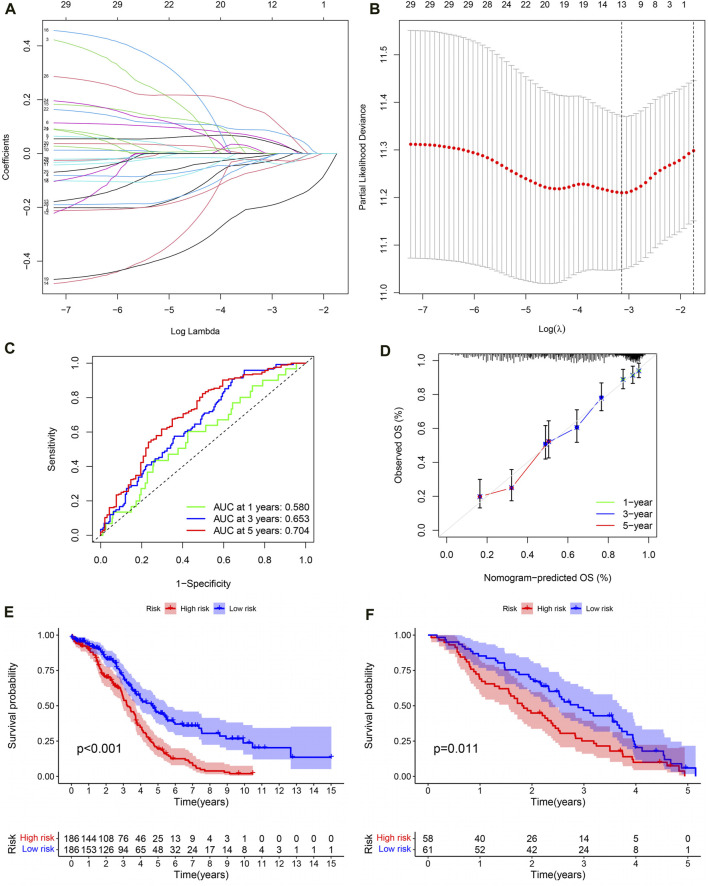
The evaluation and validation of PRSM. **(A)** Lasso regression coefficient plot. **(B)** Lasso regression parameter plot. **(C)** ROC curve of PRSM predicting prognosis. **(D)** Calibration curve of PRSM. **(E)** Kaplan–Meier analysis displays the high and low risk group OS of OC patients in TCGA. **(F)** GEO data validation of PRSM’s OS prediction ability.

Lasso regression analysis and COX regression analysis were used to screen 13 IRG signatures for building PRSM (Figures 6A, B). According to the risk score calculation formula mentioned earlier, PRSM performed a risk score on each sample and obtained high- and low-risk groups. Then, ROC curve showed that PRSM had excellent performance in predicting the prognosis of OC patients, especially the 5-year survival rate (Figure 6C, AUC = 0.704). Moreover, calibration curve indicated that the PRSM prediction results were consistent with the clinical outcomes of patients in the real world (Figure 6D). Kaplan Meier survival curve analysis revealed that the high-risk group had a shorter overall survival time compared to the low-risk group (Figure 6E, *P* < 0.001). Taking into account the low 5-year survival rate of OC patients and the accuracy of PRSM’s 5-year prediction, we conducted external validation using the GSE26712 dataset. The GSE26712 dataset contains 185 primary ovarian tumors data and 10 normal ovarian surface epithelium data. In order to verify the accuracy of the 5-year survival prediction of PRSM, 119 samples were ultimately involved in constructing the survival curve. External data has shown that PRSM has excellent performance in predicting the prognosis of OC patients (Figure 6F). We further explored whether the significantly dysregulated immune genes in ovarian cancer patients at different stages also affect treatment response or other prognostic outcomes. We found that in patients with advanced ovarian cancer, those with a high immune risk score had a worse prognosis, whereas no difference was observed in the early-stage ovarian cancer patient population (Supplementary Figures S1, S2).

### Reasons for poor prognosis in high-risk OC patients

**FIGURE 7:**
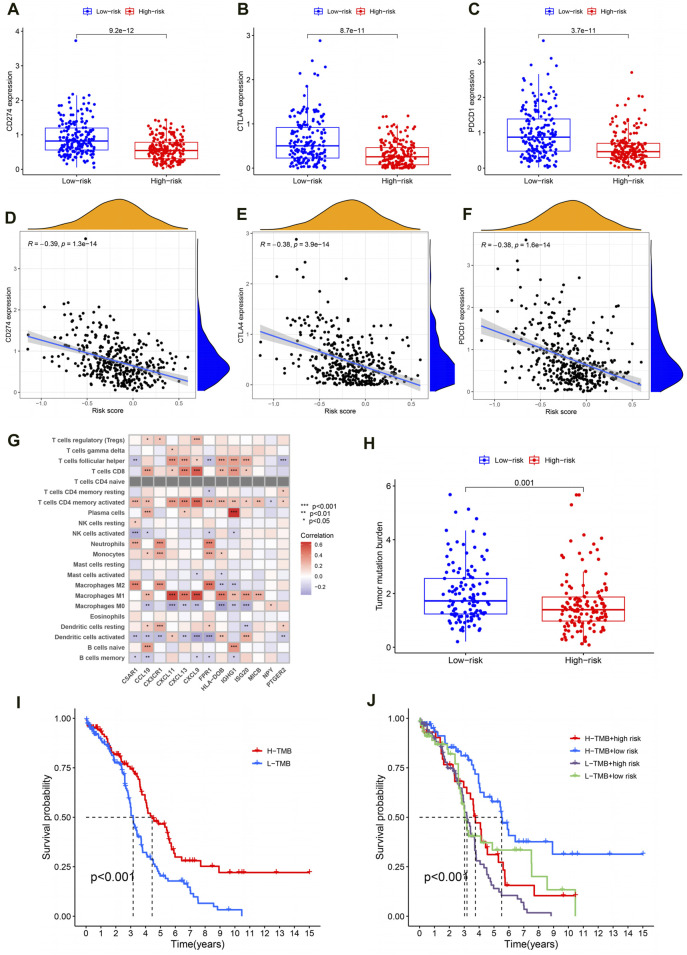
Reasons for poor prognosis in high-risk OC patients. **(A–C)** The expression level of immune checkpoint in high and low risk groups. **(D–F)** Correlation analysis between immune checkpoint expression and PRSM risk score. **(G)** Correlation analysis between IRG signatures and immune cells. **(H)** Analysis of TMB levels in high and low risk groups. **(I)** TMB level evaluation of OC prognosis in TCGA. **(J)** The PRSM risk score and TMB level jointly evaluate the prognosis of OC in TCGA.

Generally speaking, the more immune checkpoints were expressed and the higher the TMB level, the better the tumor patient’s response to immunotherapy. In order to investigate the reasons for poor prognosis in high-risk OC patients, we explored the expression levels of immune checkpoints and TMB levels. For one thing, compared to the high-risk group, the results showed lower expression of PD1 (programmed cell death protein 1, also known as CD274), CTLA4 (cytotoxic T-lymphocyte-associated protein 4) and PDL1 (programmed cell death-ligand 1, also known as PDCD1) in OC patients in the high-risk group (Figure 7A–C; *P* < 0.001). Moreover, the expression levels of these immune checkpoints are negatively correlated with the PRSM risk score (Figures 7D–F, PD1: R = −0.39, *P* = 1.3e-14; CTLA4: R = −0.38, *P* = 3.9e-14; PDL1: R = −0.38, *p* = 1.6e-14). The pearson correlation analysis between immune cells and IRG signatures showed that M2 macrophages were mainly associated with C5AR1, CX3CR1, and FPR1 (Figure 7G, *P* < 0.001). For another, TMB analysis showed that the high-risk group of OC patients had lower levels of TMB (Figure 7H, *P* = 0.001). Next, we divided the OC patients in TCGA into high- and low- TMB groups based on their TMB levels. Survival analysis suggested that patients with low TMB have a worse prognosis (Figure 7I, *P* < 0.001). Finally, we conducted survival analysis on OC patients in TCGA by combining PRSM risk score and TMB level, and the results showed that patients with low TMB level and high risk score had the worst prognosis (Figure 7J, *P* < 0.001). Therefore, it can be said that the reasons for the poor prognosis of high-risk OC patients are low expression of immune checkpoints and low TMB levels.

### Building a nomogram based on PRSM scores

**FIGURE 8:**
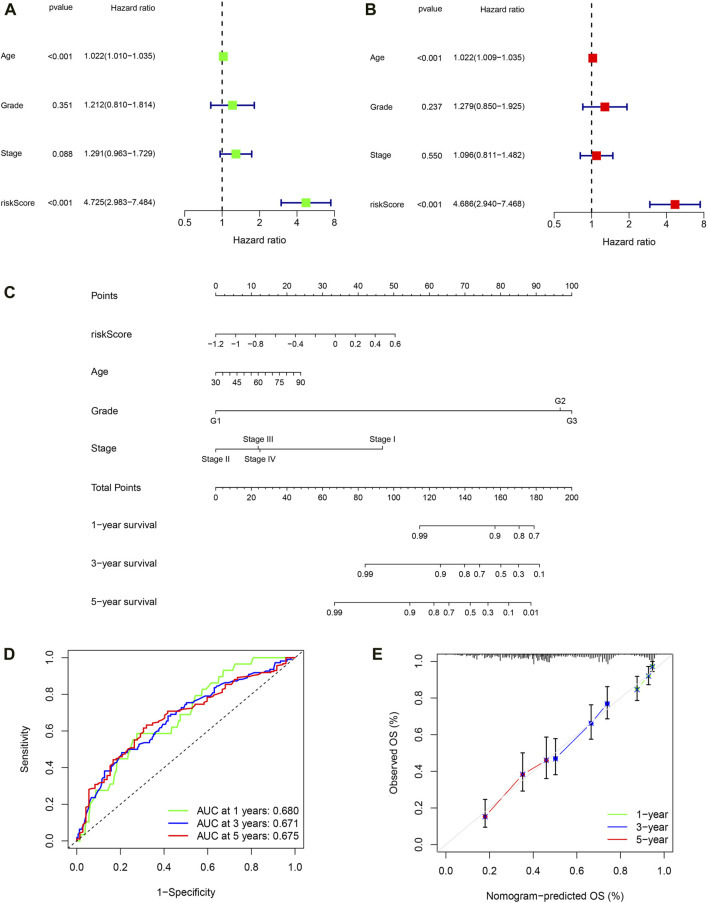
Building a nomogram based on PRSM scores. **(A)** Univariate Cox regression analyses. **(B)** Multivariate Cox regression analyses. **(C)** 1-, 3-, and 5-year OS prediction of patients with OC in TCGA through nomogram. **(D)** ROC curve of nomogram predicting prognosis. **(E)** Calibration curve of nomogram.

Firstly, univariate COX regression analysis revealed that risk score is a risk factor for the prognosis of OC patients (Figure 8A, HR: 4.725, 95% CI: 2.983–7.484, *P* < 0.001). Then, multivariate COX regression analysis confirmed that risk score is an independent risk factor for the prognosis of OC (Figure 8B, HR: 4.4.686, 95% CI: 2.940–7.468, *P* < 0.001). Next, based on patient age, tumor FIGO stage, histological grade, and PRSM risk score, we constructed a nomogram using the rsm package in the R language to predict the survival rate of OC patients (Figure 8C). ROC curve indicates that the area under the curve (AUC) predicted by nomogram for 1-year, 3-year and 5-year survival rates of OC patients is 0.680, 0.671, 0.675, respectively (Figure 8D). Predicted results of the nomogram are very close to the final clinical outcome of OC patients (Figure 8E). Hence, the nomogram has excellent predictive ability and can be used to guide clinical practice.

## Discussion

In this study, we identified two distinct immune infiltration subtypes in ovarian cancer (OC) patients using the ssGSEA method on TCGA transcriptome data, leading to the development of a prognostic risk scoring model (PRSM) based on immune-related gene (IRG) signatures. The high-immune subtype, comprising the majority of patients, presented a complex TME with both anti-tumor and immunosuppressive cells, challenging the therapeutic expectations for immunotherapy. In particular, the high-immune group exhibited lower levels of M0 macrophages and higher levels of M1 macrophages compared to the low-immune group. Our PRSM, validated on an external dataset, demonstrated robust predictive accuracy for patient prognosis, particularly for the 5-year survival rate. Furthermore, the poor prognosis in high-risk patients was associated with lower expression of immune checkpoints and reduced TMB levels, highlighting potential barriers to immunotherapy effectiveness.

TIME is one of the characteristics of tumors, moreover, the TIME of OC is even more complex (Hanahan, 2022). In ovarian cancer, tumor cells employ several strategies to evade immune surveillance. These include the downregulation of tumor antigens to reduce detection by T-cells, the secretion of immunosuppressive molecules such as TGF-β and IL-10 that create a hostile environment for immune cells, and the manipulation of immune checkpoints to inhibit T-cell activation. The immune landscape in OC is complex, with various immune cells being activated or deactivated. Tumor-associated macrophages (TAMs) often exhibit an M2-like phenotype that is associated with immune suppression and promotion of tumor growth. Regulatory T-cells (Tregs) can also be induced, further dampening the immune response. Conversely, CD8^+^ T-cells and natural killer (NK) cells are typically activated in an attempt to eliminate cancer cells, but their function may be impaired by the immunosuppressive tumor microenvironment. This is consistent with our analysis of immune cell infiltration in OC (Tian et al., 2022). On the one hand, the majority of OC patients have a large number of immune cells with anti-tumor effects in TIME, such as CD8^+^ T cells, Th1 cells, and NK cells (Matulonis et al., 2019). On the other hand, TIME of OC is rich in many immune cells with tumor-promoting or immunosuppressive effects, particularly Th2 cells and Treg cells (Savage et al., 2020). These make it challenging for immunotherapy to make breakthrough progress in OC.

Further analysis of the infiltration content of various immune cells showed that the high immune group had lower levels of M0 macrophages and higher levels of M1 macrophages than the low immune group. In the early stage of tumor formation, M1 macrophages in TIME can initiate inflammation and anti-tumor responses. However, M2 macrophages play an anti-inflammatory role and promote tumor formation. Regardless of the phenotype, macrophages are subject to complex TIME regulation (Xia et al., 2020). At present, a lot of studies have shown that promoting the polarization of tumor-associated macrophages (TAM) to M1 macrophages is conducive to improving the prognosis of colorectal cancer (Han et al., 2022), breast cancer (Asanprakit et al., 2022), and non-small cell lung cancer (Zhao B. et al., 2021). For instance, TMP195 is a selective class IIa HDAC inhibitor. TMP195 increased the proportion of M1 macrophages by promoting TAM polarization. The increase in M1 macrophages leads to an increase in the release of inflammatory cytokines, thereby enhancing the effectiveness of PD-1 blockade. In ovarian cancer, it has been shown that overexpression of lncRNA Xist regulates KLF6 through competition with miR-101 expression mediates TAM polarization towards M1 macrophages, thereby inhibiting the proliferation and migration ability of OC cells (Zhao Y. et al., 2021). On the contrary, an increase in the proportion of M2 macrophages will enhance OC chemotherapy resistance, leading to poor prognosis in OC patients (An and Yang, 2020). Furthermore, *in vitro* experiments have confirmed that paclitaxel reprogrammes M2 polarized macrophages into M1-like phenotypes in a TLR4-dependent manner, promoting the effectiveness of anti-tumor immunotherapy (Wanderley et al., 2018). Additionally, KEGG analysis and GSEA analysis showed that JAK/STAT signaling pathway was significantly enriched in OC patients, which was proved to be the critical pathways regulating M1 Macrophage polarization. Therefore, the extensive infiltration of M1 macrophages in TIME may be the key to reshaping OC’s TIME and improving immunotherapy’s efficacy. And its related regulatory pathways may be potential therapeutic targets.

In our study, the PRSM constructed based on 13 IRGs was found to has excellent prognostic prediction ability. The attenuated response to immunotherapy in the high-risk group of ovarian cancer (OC) patients might be correlated with the low expression of immune checkpoints and the decreased tumor mutation burden (TMB) observed in these patients. Conversely, patients in the low-risk group may exhibit a higher propensity to achieve clinical benefits in immunotherapy. Our results provides a new perspective for personalized treatment of OC patients in clinical practice.

Our study presents innovative findings but also has limitations that require further investigation. The role of M1 macrophages in the TIME of OC requires further *in vitro* experimental validation and mechanistic elucidation to confirm their influence on disease progression and therapeutic response. And this study did not elucidate how the differential crosstalk between tumor and non-tumor components within the tumor microenvironment might regulate the initiation and progression of the disease. Relying on TCGA and GEO databases may not fully represent the clinical diversity, suggesting a need for broader data inclusion to enhance the model’s applicability. A multicenter prospective cohort study is necessary to thoroughly assess the prognostic prediction characteristics and to validate the model’s performance across diverse patient populations. The PRSM’s predictive ability and practical utility in a clinical setting need further empirical validation to ensure it can effectively guide personalized treatment strategies. Despite these shortcomings, our research can still provide a new insight into the diagnosis and treatment of ovarian cancer patients, especially in the field of immunotherapy.

In summary, M1 macrophages and their related regulatory pathways may be potential targets for reshaping TIME and improving OC immunotherapy. Meanwhile, an effective 13-IRG marker was constructed to predict the prognosis of OC, providing new insights for immunological biomarkers.

## Data Availability

The datasets presented in this study can be found in online repositories. The names of the repository/repositories and accession number(s) can be found in the article/Supplementary Material.
