# Supplementary figures and images for: Single-sample gene set enrichment analysis reveals the clinical implications of immune-related genes in ovarian cancer
Source: Front Mol Biosci. 2024 Aug 5;11:1426274. doi: 10.3389/fmolb.2024.1426274 (PMC11330791; doi:10.3389/fmolb.2024.1426274)

# Patients with Stage III–IV

Risk + high + low

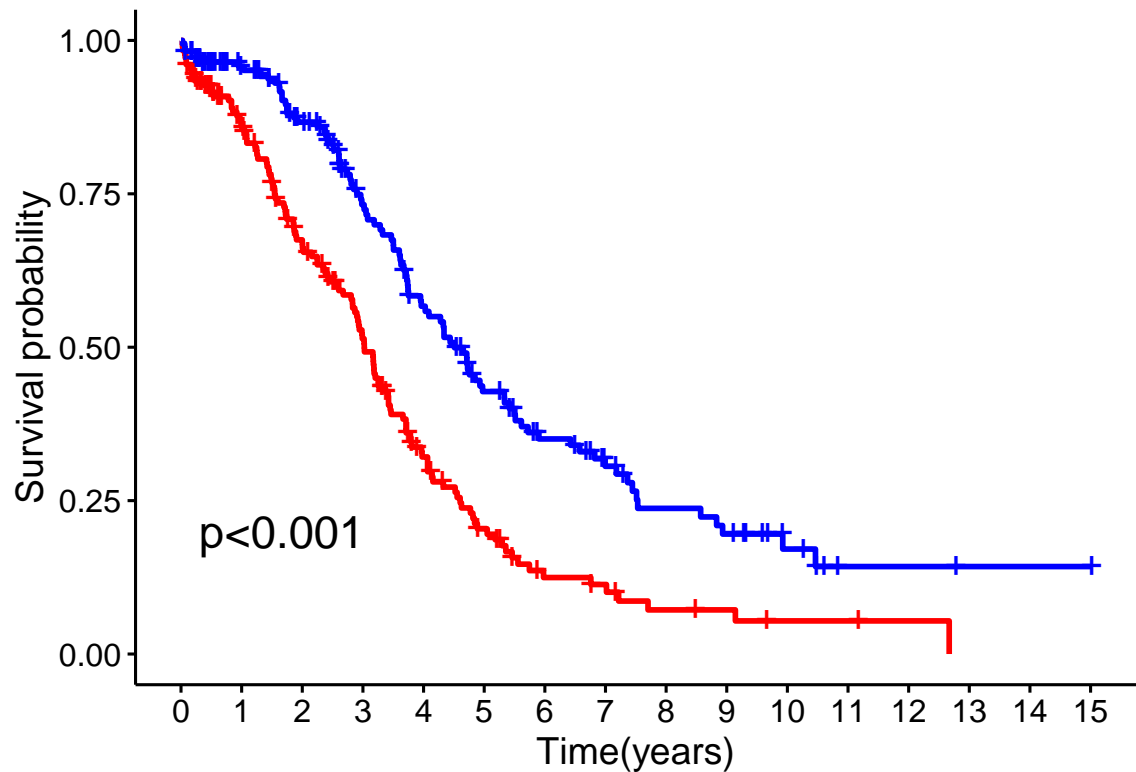

Supplement: Supplementary file 1 [file DataSheet2.PDF]

# Patients with Stage I-II

Risk + high + low

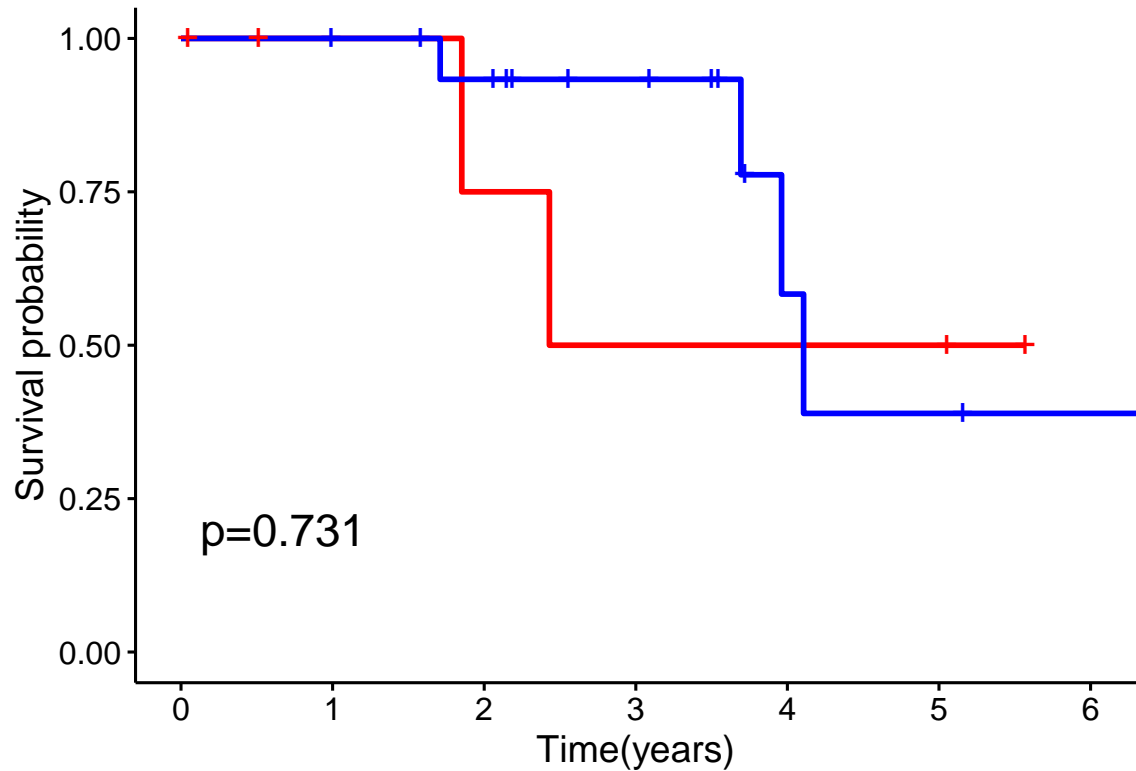

Supplement: Supplementary file 2 [file DataSheet1.PDF]
